# A Novel Virtual Reality Assessment of Functional Cognition: Validation Study

**DOI:** 10.2196/27641

**Published:** 2022-01-26

**Authors:** Lilla Alexandra Porffy, Mitul A Mehta, Joel Patchitt, Celia Boussebaa, Jack Brett, Teresa D’Oliveira, Elias Mouchlianitis, Sukhi S Shergill

**Affiliations:** 1 Institute of Psychiatry, Psychology & Neuroscience, King's College London London United Kingdom; 2 Trafford Centre for Medical Research, University of Sussex Brighton United Kingdom; 3 Faculty of Media and Communications, Bournemouth University Bournemouth United Kingdom; 4 School of Psychology, University of East London London United Kingdom; 5 Kent and Medway Medical School Canterbuy United Kingdom; 6 Kent and Medway National Heath Service and Social Care Partnership Trust Gillingham United Kingdom

**Keywords:** virtual reality, virtual reality assessment, cognition, functional cognition, functional capacity, neuropsychological testing

## Abstract

**Background:**

Cognitive deficits are present in several neuropsychiatric disorders, including Alzheimer disease, schizophrenia, and depression. Assessments used to measure cognition in these disorders are time-consuming, burdensome, and have low ecological validity. To address these limitations, we developed a novel virtual reality shopping task—VStore.

**Objective:**

This study aims to establish the construct validity of VStore in relation to the established computerized cognitive battery, Cogstate, and explore its sensitivity to age-related cognitive decline.

**Methods:**

A total of 142 healthy volunteers aged 20-79 years participated in the study. The main VStore outcomes included verbal recall of 12 grocery items, time to collect items, time to select items on a self-checkout machine, time to make the payment, time to order coffee, and total completion time. Construct validity was examined through a series of backward elimination regression models to establish which Cogstate tasks, measuring attention, processing speed, verbal and visual learning, working memory, executive function, and paired associate learning, in addition to age and technological familiarity, best predicted VStore performance. In addition, 2 ridge regression and 2 logistic regression models supplemented with receiver operating characteristic curves were built, with VStore outcomes in the first model and Cogstate outcomes in the second model entered as predictors of age and age cohorts, respectively.

**Results:**

Overall VStore performance, as indexed by the total time spent completing the task, was best explained by Cogstate tasks measuring attention, working memory, paired associate learning, and age and technological familiarity, accounting for 47% of the variance. In addition, with *λ*=5.16, the ridge regression model selected 5 parameters for VStore when predicting age (mean squared error 185.80, SE 19.34), and with *λ*=9.49 for Cogstate, the model selected all 8 tasks (mean squared error 226.80, SE 23.48). Finally, VStore was found to be highly sensitive (87%) and specific (91.7%) to age cohorts, with 94.6% of the area under the receiver operating characteristic curve.

**Conclusions:**

Our findings suggest that VStore is a promising assessment that engages standard cognitive domains and is sensitive to age-related cognitive decline.

## Introduction

### Background

Cognitive dysfunction refers to deficits in intellectual functions usually described by domains such as attention, working memory, verbal and visual learning, executive function, and processing speed. Deficits in cognition are evident across a range of neuropsychiatric disorders, including Alzheimer disease (AD), schizophrenia, and depression. Although these intellectual deficits are diagnostic in AD [1], 90% of individuals with schizophrenia and depression are also affected [2,3]. This is further complicated by the observation that some cognitive decline is part of the natural aging process and is reported in one-quarter of older adults without dementia [4]. The high prevalence of cognitive dysfunction in mental and physical [5] illness, an increasingly aging population, and the lack of robust treatments suggest that the global burden of cognitive dysfunction has a substantial socioeconomic impact.

Cognitive decline has a marked effect on functional recovery and quality of life in patients with mental disorders [6-8]. In addition, it precedes and predicts functional outcomes in AD and schizophrenia [9,10] and predicts treatment response in depression [11], highlighting the urgent need to effectively target these symptoms. Unfortunately, clinical trials of cognitive enhancers have been largely disappointing [12-14]. Indeed, most compounds that demonstrated positive effects in phase 2 trials have failed in phase 3 trials. This raises several questions about the sensitivity of our cognitive assessments and the targets of these interventions.

Standard cognitive assessments are designed to evaluate changes in distinct neuropsychological domains, whereas the actual target for therapy is change in functional cognition—the ability to perform everyday routine activities [15]. Accordingly, the Food and Drug Administration has mandated the assessment of real-life functional change, alongside changes in conventional cognitive performance, as a condition for drug approval for both AD and schizophrenia [16,17]. This is particularly important as there can be a lack of concordance between changes in cognitive measures and related everyday functioning. For example, cognitive task performance only explains 20% of the variance in work-related skills in schizophrenia [18]. Although there has been an attempt to supplement cognitive assessments with self-report and reports by caregivers to assess wider functioning, these assessments lack objectivity.

A related issue is that cognitive assessments require optimal task engagement, which can be confounded by poor attention and motivation [19,20]. The gold standard cognitive measure for AD, the Alzheimer’s Disease Assessment Scale–Cognitive Subscale [21], takes approximately 45 minutes to administer; the analogous scale for schizophrenia, the MATRICS Consensus Cognitive Battery [22,23], takes up to 90 minutes. The related functional capacity assessment for AD, the Clinical Dementia Rating Scale [24], takes approximately 30 minutes to complete, similar to the University of California, San Diego Performance-Based Skills Assessment [25] for schizophrenia. The ecological validity and predictive power of real-life performance of standard assessments have also been questioned [26,27].

Complex assessments that emulate everyday scenarios have been developed, including the Multiple Errands Test (MET), which measures executive function in patients with traumatic brain injury [28], and the Virtual Reality Functional Capacity Assessment Tool (VRFCAT), which measures functional skills in schizophrenia and can reliably differentiate patients from controls [29,30]. However, the MET is time-consuming and difficult to standardize with a lack of experimental control, whereas the VRFCAT lacks full ecological validity as it is completed on a computer or tablet without the immersive nature of real-life interactions.

### Objectives

Recent developments in technology, specifically in virtual reality (VR), now enable us to create assessments that can replicate challenges found in everyday life while also maintaining experimental control [31]. This offers the opportunity to overcome issues associated with current assessments. In this study, we describe the development of a novel, fully immersive VR assessment, VStore, with the aim to simultaneously assess traditional cognitive domains and functional capacity. This is achieved through the creation of an ecologically valid minimarket environment with a maze-like layout. Each action within the assessment maps an embedded cognitive task (eg, recall of shopping list items measures verbal memory), and each task is assessed by performing actions that require almost identical procedures similar to shopping in real life, offering a measure of concurrent functional capacity. Moving in an immersive VR environment engages brain structures associated with spatial navigation, such as the hippocampus and entorhinal cortex [32], which are affected in early AD [33], depression [34], and schizophrenia [35]. Therefore, VStore may be more sensitive to early neurodegenerative processes than the existing assessments.

The aim of this study is 2-fold. First, we establish the cognitive domains relevant to VStore performance. More specifically, we test which cognitive processes, as measured by an existing standard cognitive battery, predict VStore performance as an initial evaluation of its construct. We achieve this by conducting a series of stepwise prediction models. Second, we explore the preliminary utility of VStore in assessing cognitive decline associated with nonpathological aging. This is achieved by testing VStore’s ability to predict age both as a continuous and dichotomized outcome.

## Methods

### Participants

A total of 142 healthy volunteers aged 20-79 years were recruited through advertisements in college circular emails, charity newsletters, and social media. Participants were excluded if they had (1) a diagnosis of an axis 1 disorder (Diagnostic and Statistical Manual of Mental Disorders, 5th edition); (2) dependence on alcohol or illicit substances; (3) clinically significant motion sickness; (4) a pregnancy; and (5) a diagnosis of a neurological illness. Of the 142 volunteers, 38 (26.8%) participants were excluded from the study. The reasons for exclusion were as follows: 1 participant withdrew consent, 1 could not complete VStore owing to technical issues, 1 senior participant could not complete VStore owing to fatigue, 20 participants failed either or both integrity and completion criteria for Cogstate, and 15 participants were removed owing to outlier values on one or more primary outcome measures. The demographic information for the final sample of 73.2% (104/142) of participants is presented in Table 1.

**Table 1:** Sample demographics.

Variable	Age group (years)						
	20-29	30-39	40-49	50-59	60-69	70-79	Total
Population, n (%)	19 (18.3)	18 (17.3)	18 (17.3)	17 (16.3)	18 (17.3)	14 (13.5)	104 (100)
Age (years), mean (SD; range)	23.6 (2.5; 20-29)	32.7 (2.3; 30-37)	45.2 (2.8; 40-49)	53.5 (3.0; 50-59)	64.2 (2.4; 61-69)	73.4 (3.3; 70-79)	48.8 (17.8; 20-79)
Gender (female), n (%)	10 (52.6)	11 (61.1)	9 (50)	8 (47.1)	10 (55.6)	6 (42.9)	54 (51.9)
IQ, mean (SD; range)	119.7 (9.5; 96-136)	121.3 (6.9; 105-131)	120.6 (6.0; 106-132)	121.7 (7.9; 109- 133)	126.3 (5.9; 113-134)	128.1 (6.3; 118-138)	122.9 (10.3; 96-138)
Education (years); mean (SD; range)	17.6 (2.4; 15-24)	19.4 (3.0; 14-27)	18.8 (1.9; 15-22)	18.0 (9.5; 11-20)	16.1 (3.4; 10-23)	18.1 (5.3; 10-25)	18.0 (3.6; 10-27)
Technological familiarity, mean (SD; range)	47.1 (6.8; 34-57)	47.2 (6.4; 32-57)	43.5 (7.9; 32-58)	43.4 (7.9; 28-57)	35.6 (8.3; 21-52)	33.4 (8.6; 21-48)	41.7 (9.7; 21-58)

### Measures

#### VStore

VStore was developed in collaboration with Vitae VR [36]. It takes approximately 30 minutes to complete, including orientation, instructions, practice, and assessment. Orientation and practice are set in a courtyard specifically designed for VR acclimatization (Multimedia Appendix 1).

The assessment itself is set in a minimarket environment depicting a fruit and vegetable section; 6 aisles of foodstuff, snacks, drinks, and toiletries; fridges with chilled drinks and sandwiches; and freezers with frozen meals. In addition, there are checkout and self-checkout counters and a coffee shop at the back of the minimarket. A total of 66 items, organized into 9 categories, were created to fill the shop (Multimedia Appendix 2).

**Figure 1:**
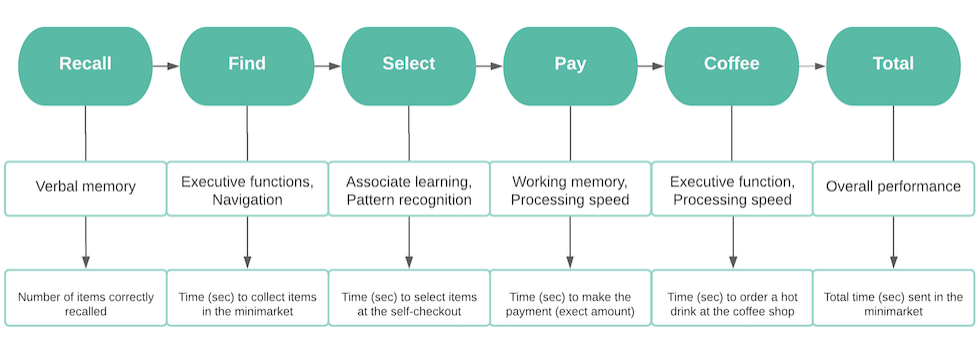
Flowchart depicting the steps required to complete VStore, its corresponding cognitive domains, and outcome variables.

At the start, participants were read out 12 items from a shopping list (Multimedia Appendix 3) by the avatar standing near the entrance. The first task of the participants was to memorize and recall as many items from this list as possible. Following recall, participants were presented with the shopping list, including all 12 items, and instructed to move around the shop and collect all items as quickly and accurately as possible. Once all the items are bagged, they are required to select and pay for them at a self-checkout machine, providing the exact amount (Multimedia Appendix 4). The task is concluded when participants order a hot drink from the coffee shop situated in the minimarket. Progression to the next task could only be achieved after successfully completing the previous task. The steps required to complete the VStore tasks are summarized in Figure 1. Multimedia Appendices 5-7 provide details on apparatus information, software information, and how movement is executed in the virtual environment, respectively.

#### Cogstate

Cogstate is a computerized cognitive battery designed to assess multiple cognitive domains. It has been widely used in both healthy and clinical populations. Cogstate is simple to use, even for adults with limited computer experience, and therefore suitable for testing older adults [37]. For the purposes of this study, 8 tasks that cover key cognitive domains (Table 2 and Multimedia Appendix 8) were selected, taking approximately 30-40 minutes to complete.

**Table 2:** List of Cogstate tasks, corresponding cognitive domains assessed, and main outcome measures.

Code	Task name	Cognitive domain	Outcome (metric)
DET	Detection	Processing speed	Reaction time (log 10 ms)
IDN	Identification	Attention	Reaction time (log 10 ms)
OCL	One Card Learning	Visual learning	Accuracy (arcsine proportion)
ONB	One-Back	Working memory	Reaction time (log 10 ms)
TWO	Two-Back	Working memory	Accuracy (arcsine proportion)
CPAL	Continuous Paired Associate Learning	Paired associate learning	Total number of errors (N/A^a^)
GMLT	Groton Maze Learning	Executive functions	Total number of errors (N/A)
ISLT	International Shopping List Task	Verbal learning	Number of correct responses (N/A)

^a^N/A: not applicable.

#### Wechsler Abbreviated Scale of Intelligence

The abbreviated version of the Wechsler Adult Intelligence Scale was used to establish the IQ of participants [38]. Specifically, the 2-item scale included matrix and vocabulary tests.

#### Technological Familiarity Questionnaire

We developed a self-report questionnaire to assess the technological familiarity of the sample population. Participants were asked 13 questions to ascertain their frequency, comfort, and ability in technology use. Higher scores indicated more technological familiarity. The internal consistency of the questionnaire was good (Cronbach α=.88). A detailed description of the Technological Familiarity Questionnaire (TFQ) is presented in Multimedia Appendix 9.

### Procedures

Potential participants were prescreened over the phone. If they were deemed eligible, they were invited for a single study visit that lasted up to 2.5 hours. First, informed consent was obtained, followed by obtaining demographics, brief mental and physical health history, and the TFQ scores. Cogstate and VStore were administered in a counterbalanced fashion to mitigate any order effects. All the participants received the same shopping list. Finally, the Wechsler Abbreviated Scale of Intelligence was administered. Participants were compensated for their time and reimbursed for travel expenses. Ethical approval was granted by the Psychiatry, Nursing and Midwifery Research Ethics Committee, King’s College London (LRS-16/17-4540).

### Analysis

Before data analysis, VStore outcome variables measured in seconds were log-transformed to stabilize the variance. Descriptive statistics for both VStore and Cogstate outcomes are presented in Multimedia Appendices 10 and 11. As an initial overview of the relationship between Cogstate and VStore, Bonferroni-corrected Spearman ρ was calculated between the 2 assessments. These results are presented in Multimedia Appendix 12.

To establish which cognitive domains, assessed by Cogstate, best predicted VStore performance, we ran a series of backward elimination regression models implemented in the R package MASS [39]. VStore outcomes were entered as dependent variables (DVs) and all 8 Cogstate tasks were entered as independent variables (IVs). Age and technological familiarity (TFQ) were also entered as IVs, as these (but not IQ) showed a significant relationship with VStore outcomes. All IVs were standardized using the sample mean and SD to create *z* scores. Regression models were penalized for complexity using the Akaike Information Criterion (AIC) to arrive at the most parsimonious model. Additional quality checks for the final models are presented in Multimedia Appendix 13. These confirm that the assumptions of normality and homoscedasticity were met.

As an exploratory objective to examine the potential of VStore in predicting age, we used ridge regression, implemented in the R package glmnet [40], where regularization is governed by 2 parameters— α and *λ*. We set the penalty parameter, α, to 0 (to enforce ridge regression, where the estimated coefficients of strongly correlated predictor variables are *shrunk* toward each other). The optimal value of the strength of this penalty (*λ*) was determined using leave-one-out cross-validation (ie, for a given value of *λ*, training on N-1 participants, and testing performance on the one participant who is held-out by computing the mean squared error [MSE]). The DV was age for 104 participants. In the first model, IVs included all VStore outcomes except for total time: Recall, Find, Select, Pay, and Coffee. In the second model, IVs included all Cogstate tasks: Detection (DET), Identification (IDN), One Card Learning (OCL), One-Back (ONB), Two-Back (TWO), Continuous Paired Associate Learning (CPAL), Groton Maze Learning (GMLT), and the International Shopping List Task (ISLT). Both models were repeated with technological familiarity (TFQ) included as an additional IV to indicate whether VStore was confounded by technological familiarity. Finally, to further probe VStore’s sensitivity in predicting age cohorts, we took the top and bottom 20% of the sample population based on age and ran 2 logistic regression models to generate 2 overlying receiver operating characteristic curves—one for VStore and one for Cogstate. The bottom fraction of the sample included 23.1% (24/104) of participants aged 20-30 years, whereas the top fraction included 22.1% (23/104) of participants aged 65-79 years. Similar to the regression analyses, the age cohort (0, 1) was entered as the DV, and IVs for VStore model were Recall, Find, Select, Pay, and Coffee, whereas the IVs for the Cogstate model included DET, IDN, OCL, ONB, TWO, CPAL, GMLT, and ISLT. Youden *J* statistic was used to establish the optimal threshold for sensitivity and specificity, and model performance was compared with the DeLong test.

## Results

### VStore Construct

Tables 3-5 summarizes the predictors of VStore performance. The initial model included all Cogstate variables, in addition to age and technological familiarity. Backward elimination regression resulted in the removal of several of these predictors, without any substantial change in the variance explained by the models. AIC values showed a decrease from the initial to final models, arriving at a more parsimonious set of predictors for each VStore outcome.

Recalling items from VStore shopping list was predicted by verbal learning. Finding items in VStore was best explained by attention (IDN), working memory (ONB), paired associate learning (CPAL), age, and technological familiarity (TFQ). The best predictors of VStore Select were working memory (TWO), executive functions (GMLT), verbal learning (ISLT), and age. Paying for items in VStore was best explained by processing speed (DET), working memory (TWO), executive function (GMLT), verbal learning (ISLT), and technological familiarity (TFQ). Time to order a coffee was best predicted by visual (OCL) and verbal (ISLT) learning, working memory (TWO), and age. Finally, total time spent in VStore was best explained by attention (IDN), working memory (ONB), paired associate learning (CPAL), age, and technological familiarity (TFQ). For the final model, the explained variance ranged from 25% for VStore Select to 47% for VStore Total time.

Given the prominent role of technological familiarity in VStore performance, we also examined the correlations between the TFQ and Cogstate for comparison. Indeed, 6 out of 8 Cogstate tasks (DET, IDN, ONB, TWO, CPAL, and GMLT) had a significant relationship with the TFQ (Multimedia Appendix 14).

**Table 3:** Initial and final linear regression models examining the construct validity of VStore for Recall, Find, and Select outcomes.

DV^a^ and IV^b^			Initial model											Final model								
			B (SE)		*P* value		*R* ^2^		*F* test (*df*)		AIC^c^		B (SE)			*P* value		*R* ^2^	*F* test (*df*)			AIC
**Recall**																						
	DET^d^	0.264 (0.205)		.20		N/A^e^		N/A		N/A		N/A			N/A		N/A			N/A	N/A	
	IDN^f^	−0.064 (0.224)		.78		N/A		N/A		N/A		N/A			N/A		N/A			N/A	N/A	
	OCL^g^	0.078 (0.190)		.68		N/A		N/A		N/A		N/A			N/A		N/A			N/A	N/A	
	ONB^h^	−0.016 (0.520)		.95		N/A		N/A		N/A		N/A			N/A		N/A			N/A	N/A	
	TWO^i^	0.246 (0.218)		.26		N/A		N/A		N/A		N/A			N/A		N/A			N/A	N/A	
	GMLT^j^	−0.056 (0.214)		.79		N/A		N/A		N/A		N/A			N/A		N/A			N/A	N/A	
	CPAL^k^	0.209 (0.214)		.33		N/A		N/A		N/A		N/A			N/A		N/A			N/A	N/A	
	ISLT^l^	0.569 (0.202)		.006		N/A		N/A		N/A		0.056 (0.014)			<.001		N/A			N/A	N/A	
	Age	−0.312 (0.242)		.20		N/A		N/A		N/A		N/A			N/A		N/A			N/A	N/A	
	TFQ^m^	0.001 (0.228)		.95		.10		2.138 (10, 93)		123.6		N/A			N/A		.13			16.210^n^ (1, 102)	111.8	
**Find**																						
	DET	0.019 (0.028)		.047		N/A		N/A		N/A		N/A			N/A		N/A			N/A	N/A	
	IDN	−0.061 (0.030)		.93		N/A		N/A		N/A		−0.052 (0.028)			.07		N/A			N/A	N/A	
	OCL	−0.002 (0.026)		.06		N/A		N/A		N/A		N/A			N/A		N/A			N/A	N/A	
	ONB	0.065 (0.034)		.92		N/A		N/A		N/A		0.074 (0.030)			.03		N/A			N/A	N/A	
	TWO	−0.003 (0.029)		.40		N/A		N/A		N/A		N/A			N/A		N/A			N/A	N/A	
	GMLT	0.024 (0.029)		.11		N/A		N/A		N/A		N/A			N/A		N/A			N/A	N/A	
	CPAL	0.046 (0.029)		.84		N/A		N/A		N/A		0.055 (0.025)			.03		N/A			N/A	N/A	
	ISLT	−0.006 (0.027)		.006		N/A		N/A		N/A		N/A			N/A		N/A			N/A	N/A	
	Age	0.093 (0.033)		.03		N/A		N/A		N/A		0.100 (0.029)			.001		N/A			N/A	N/A	
	TFQ	−0.070 (0.031)		.50		.40		7.833^n^ (10, 93)		−293.1		−0.068 (0.028)			.02		.42			15.830^n^ (5, 98)	−301.1	
**Select**																						
	DET	−0.006 (0.034)		.86		N/A		N/A		N/A		N/A			N/A		N/A			N/A	N/A	
	IDN	−0.035 (0.037)		.35		N/A		N/A		N/A		N/A			N/A		N/A			N/A	N/A	
	OCL	−0.009 (0.031)		.77		N/A		N/A		N/A		N/A			N/A		N/A			N/A	N/A	
	ONB	0.020 (0.041)		.64		N/A		N/A		N/A		N/A			N/A		N/A			N/A	N/A	
	TWO	−0.034 (0.036)		.35		N/A		N/A		N/A		−0.043 (0.030)			.16		N/A			N/A	N/A	
	GMLT	0.041 (0.035)		.252		N/A		N/A		N/A		0.043 (0.031)			.16		N/A			N/A	N/A	
	CPAL	0.024 (0.035)		.50		N/A		N/A		N/A		N/A			N/A		N/A			N/A	N/A	
	ISLT	−0.041 (0.033)		.22		N/A		N/A		N/A		−0.047 (0.030)			.12		N/A			N/A	N/A	
	Age	0.111 (0.040)		.007		N/A		N/A		N/A		0.108 (0.030)			.001		N/A			N/A	N/A	
	TFQ	0.001 (0.038)		.97		.22		3.896^n^ (10, 93)		−251.6		N/A			N/A		.25			9.783^n^ (4, 99)	−261.8	

^a^DV: dependent variable.

^b^IV: independent variable.

^c^AIC: Akaike Information Criterion.

^d^DET: Detection (processing speed).

^e^N/A: not applicable.

^f^IDN: Identification (attention).

^g^OCL: One Card Learning (visual learning).

^h^ONB: One-Back (working memory).

^i^TWO: Two-Back (working memory).

^j^GMLT: Groton Maze Learning (executive function).

^k^CPAL: Continuous Paired Associate Learning (paired associate learning).

^l^ISLT: International Shopping List Task (Verbal learning).

^m^TFQ: Technological Familiarity Questionnaire.

^n^Significant at *P*<.001.

**Table 4:** Initial and final linear regression models examining the construct validity of VStore for Pay and Coffee outcomes.

DV^a^ and IV^b^			Initial model											Final model								
			B (SE)		*P* value		*R* ^2^		*F* test (*df*)		AIC^c^		B (SE)			*P* value		*R* ^2^		*F* test (*df*)		AIC
**Pay**																						
	DET^d^	0.043 (0.040)		.28		N/A^e^		N/A		N/A		0.063 (0.035)			.08		N/A		N/A		N/A	
	IDN^f^	0.024 (0.043)		.59		N/A		N/A		N/A		N/A			N/A		N/A		N/A		N/A	
	OCL^g^	0.032 (0.037)		.38		N/A		N/A		N/A		N/A			N/A		N/A		N/A		N/A	
	ONB^h^	0.034 (0.048)		.49		N/A		N/A		N/A		N/A			N/A		N/A		N/A		N/A	
	TWO^i^	−0.085 (0.042)		.047		N/A		N/A		N/A		−0.085 (0.036)			.02		N/A		N/A		N/A	
	GMLT^j^	0.070 (0.041)		.09		N/A		N/A		N/A		0.060 (0.037)			.11		N/A		N/A		N/A	
	CPAL^k^	−0.025 (0.041)		.55		N/A		N/A		N/A		N/A			N/A		N/A		N/A		N/A	
	ISLT^l^	−0.072 (0.039)		.07		N/A		N/A		N/A		−0.077 (0.034)			.03		N/A		N/A		N/A	
	Age	0.020 (0.047)		.67		N/A		N/A		N/A		N/A			N/A		N/A		N/A		N/A	
	TFQ^m^	−0.106 (0.044)		.02		.34		6.409^n^ (10, 93)		−218.6		−0.129 (0.035)			<.001		.36		12.630^n^ (5, 98)		−225.8	
**Coffee**																						
	DET	0.018 (0.048)		.72		N/A		N/A		N/A		N/A			N/A		N/A		N/A		N/A	
	IDN	−0.018 (0.053)		.73		N/A		N/A		N/A		N/A			N/A		N/A		N/A		N/A	
	OCL	0.075 (0.045)		.10		N/A		N/A		N/A		0.077 (0.041)			.07		N/A		N/A		N/A	
	ONB	0.021 (0.059)		.72		N/A		N/A		N/A		N/A			N/A		N/A		N/A		N/A	
	TWO	−0.046 (0.051)		.37		N/A		N/A		N/A		−0.078 (0.042)			.07		N/A		N/A		N/A	
	GMLT	−0.003 (0.051)		.96		N/A		N/A		N/A		N/A			N/A		N/A		N/A		N/A	
	CPAL	0.061 (0.051)		.23		N/A		N/A		N/A		N/A			N/A		N/A		N/A		N/A	
	ISLT	−0.060 (0.048)		.21		N/A		N/A		N/A		−0.066 (0.044)			.13		N/A		N/A		N/A	
	Age	0.181 (0.057)		.002		N/A		N/A		N/A		0.218 (0.043)			<.001		N/A		N/A		N/A	
	TFQ	−0.027 (0.054)		.62		.27		4.751^n^ (10, 93)		−177.0		N/A			N/A		.30		11.810^n^ (4, 99)		−186.6	

^a^DV: dependent variable.

^b^IV: independent variable.

^c^AIC: Akaike Information Criterion.

^d^DET: Detection (processing speed).

^e^N/A: not applicable.

^f^IDN: Identification (attention).

^g^OCL: One Card Learning (visual learning).

^h^ONB: One-Back (working memory).

^i^TWO: Two-Back (working memory).

^j^GMLT: Groton Maze Learning (executive function).

^k^CPAL: Continuous Paired Associate Learning (paired associate learning).

^l^ISLT: International Shopping List Task (Verbal learning).

^m^TFQ: Technological Familiarity Questionnaire.

^n^Significant at *P*<.001.

**Table 5:** Initial and final linear regression models examining the construct validity of VStore Total.

DV^a^ and IV^b^		Initial model						Final model				
		B (SE)	*P* value	*R* ^2^	*F* test (*df*)	AIC^c^	B (SE)		*P* value	*R* ^2^	*F* test (*df*)	AIC
**Total**												
	DET^d^	0.018 (0.020)	.44	N/A^e^	N/A	N/A	N/A		N/A	N/A	N/A	N/A
	IDN^f^	−0.046 (0.024)	.08	N/A	N/A	N/A	−0.039 (0.025)		.12	N/A	N/A	N/A
	OCL^g^	0.003 (0.026)	.88	N/A	N/A	N/A	N/A		N/A	N/A	N/A	N/A
	ONB^h^	0.048 (0.022)	.10	N/A	N/A	N/A	0.067 (0.026)		.01	N/A	N/A	N/A
	TWO^i^	−0.015 (0.029)	.56	N/A	N/A	N/A	N/A		N/A	N/A	N/A	N/A
	GMLT^j^	0.025 (0.025)	.32	N/A	N/A	N/A	N/A		N/A	N/A	N/A	N/A
	CPAL^k^	0.043 (0.025)	.09	N/A	N/A	N/A	0.059 (0.021)		.007	N/A	N/A	N/A
	ISLT^l^	−0.023 (0.025)	.34	N/A	N/A	N/A	N/A		N/A	N/A	N/A	N/A
	Age	0.097 (0.024)	.001	N/A	N/A	N/A	0.109 (0.025)		<.001	N/A	N/A	N/A
	TFQ^m^	−0.050 (0.028)	.06	.46	9.803^n^ (10, 93)	−323.3	−0.045 (0.025)		.07	.47	19.070^n^ (5, 98)	−329.1

^a^DV: dependent variable.

^b^IV: independent variable.

^c^AIC: Akaike Information Criterion.

^d^DET: Detection (processing speed).

^e^N/A: not applicable.

^f^IDN: Identification (attention).

^g^OCL: One Card Learning (visual learning).

^h^ONB: One-Back (working memory).

^i^TWO: Two-Back (working memory).

^j^GMLT: Groton Maze Learning (executive function).

^k^CPAL: Continuous Paired Associate Learning (paired associate learning).

^l^ISLT: International Shopping List Task (Verbal learning).

^m^TFQ: Technological Familiarity Questionnaire.

^n^Significant at *P*<.001.

### Cognitive Performance as Predictor of Age

**Figure 2:**
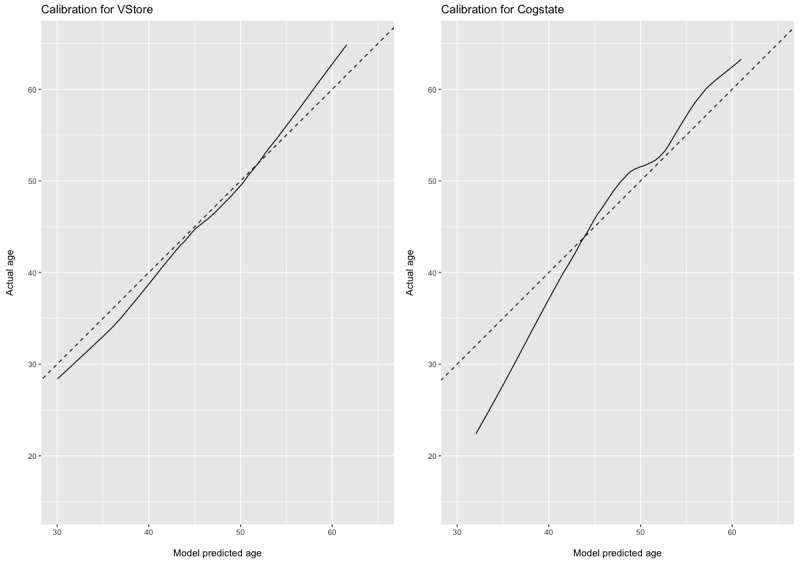
VStore and Cogstate models predicting age. Dashed lines depict the age of the participants. The solid stroke shows the age predicted by the ridge regression models.

For the DV age, we built 2 models using VStore and Cogstate outcomes as predictors (Figure 2). In the VStore model, the model fitting achieved an MSE of 185.8 (SE 19.34), selecting a total of 5 predictors and from cross-validating, which was attained from an optimal *λ* of 5.16 (Multimedia Appendix 15). For the Cogstate model, we found an MSE of 226.8 (SE 23.48), selecting a total of 8 predictors obtained with an optimal *λ* of 9.49 (Multimedia Appendix 16). We also fitted a null (intercept-only) model that yields an MSE of 294.71, suggesting that models for both VStore and Cogstate are preferable to a model with no predictors.

In the VStore model, coefficient values were as follows: VStore Recall=−0.586; VStore Find=7.882; VStore Select=5.284; VStore Pay=3.291; and VStore Coffee=4.526. In this model, the Find task is most strongly positively associated with increasing age, followed by the Select, Coffee, Pay, and Recall tasks.

In the Cogstate model, the coefficient values were as follows: DET=11.089; IDN=15.277; OCL=−2.563; ONB=12.038; TWO=−1.293; GMLT=0.032; CPAL=0.015; and ISLT=−0.245. In this model, the IDN task was most strongly positively associated with increasing age, followed by the ONB, DET, and TWO tasks.

**Figure 3:**
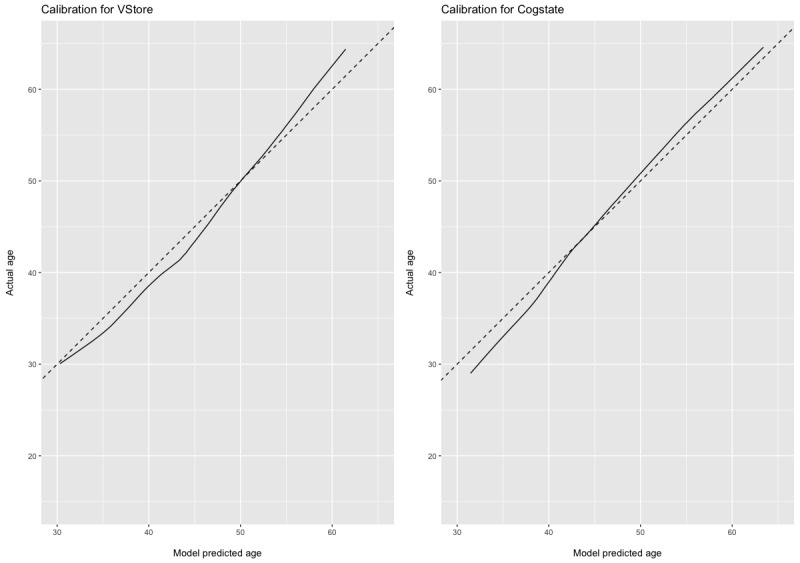
VStore and Cogstate models predicting age with technological familiarity included. Dashed lines depict the age of the participants. The solid stroke shows the age predicted by the ridge regression models.

For the DV age, we built 2 additional models using VStore and Cogstate outcomes as predictors with technological familiarity included as a covariate (Figure 3). With the TFQ added to the VStore model, the model fitting achieved an MSE of 162.9 (SE 17.50), selecting a total of 6 predictors and from cross-validating, this was attained from an optimal *λ* at 3.904 (Multimedia Appendix 17). With the TFQ added to the Cogstate model, we found an MSE of 175.4 (SE 22.12), selecting a total of 9 predictors obtained with an optimal *λ* at 2.904 (Multimedia Appendix 18).

In the VStore model, coefficient values were as follows: VStore Recall=−0.617; VStore Find=7.298; VStore Select=5.422; VStore Pay=2.699; VStore Coffee=4.517, and TFQ=−0.279.

In the Cogstate model, coefficient values were as follows: DET=16.777; IDN=22.388; OCL=−3.544; ONB=15.451; TWO=2.034; GMLT=0.0438; CPAL=0.0243, ISLT=−0.479; and TFQ=−0.348.

### Age Cohort Classification

**Figure 4:**
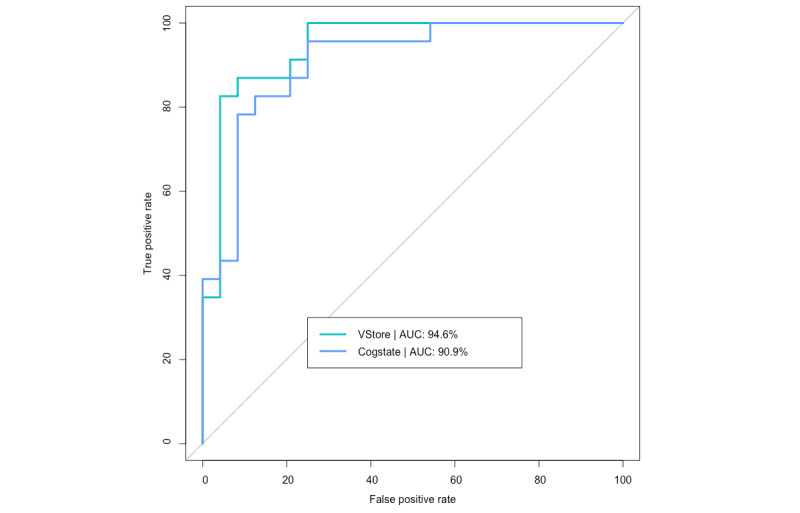
VStore and Cogstate models predicting age cohorts. AUC: area under the receiver operating characteristic curve.

Figure 4 shows the sensitivity of the VStore and Cogstate models in classifying age cohorts of 20-30 and 65-79 years of this study’s sample population. VStore has a sensitivity of 87% and specificity of 91.7% at the optimal threshold of 0.55, whereas Cogstate has a sensitivity of 95.7% and specificity of 75% at the optimal threshold of 0.36. The difference between the 2 models was not statistically significant (*Z*=0.69, *P*=.49).

## Discussion

### Principal Findings

The primary aim of this study was to establish which cognitive functions are engaged during a novel VR assessment, VStore. We found that a number of cognitive processes, as measured by Cogstate, contributed to the variance explained in VStore performance, suggesting that the VR task engages a range of key neuropsychological functions simultaneously. Indeed, the realistic nature of VStore precludes a simple one-to-one mapping between Cogstate domains and VStore outcomes. These findings provide preliminary information about VStore’s construct validity and show that functional tasks embedded in VR may engage a greater range of cognitive domains than standard assessments because of their increased complexity and ability to resemble the demands of the real world [41].

As anticipated, VStore Recall was best explained by the Cogstate verbal learning task. VStore Find demonstrated a significant relationship with a number of predictors including attention, working memory, paired associate learning, age, and technological familiarity. VStore Select was explained by working memory, executive function, verbal learning, and technological familiarity. The more items participants could remember, the quicker they selected them on the self-checkout machine (verbal learning); attentional control (executive function) and temporary memorization of remaining items (working memory) were also required. VStore Pay engaged working memory, executive functions, and required processing speed. VStore Coffee was explained by visual and verbal learning, working memory, and age. Finally, the total time spent in VStore was best explained by Cogstate tasks measuring paired associate learning and working memory, in addition to the participants’ age and technological familiarity, accounting for almost half of the variance in VStore performance.

The CPAL task of Cogstate is an episodic memory paradigm that involves visuospatial processing and indexes the ability to learn, store, and retrieve information. Paired associations may be especially important when finding items in a store, as this requires the retrieval of object representations from the shopping list, such as Cornflakes. Severe impairment in this domain has been linked to a number of neuropsychiatric conditions, including AD [42], and has been shown to be a valuable tool for the early detection of the disorder [43]. Deficits in paired associate learning have also been observed in schizophrenia and are linked to hippocampal volume loss [44].

Working memory, the temporary retention of information for manipulation and decision-making, is a key cognitive process in overall VStore performance. It is particularly relevant for the stages of the assessment where reviewing the shopping list is necessary to successfully carry out the next step of the task, such as finding an item or selecting it on the self-checkout machine. In support of the role of working memory in complex cognitive and functional assessments, factor analysis revealed that working memory was one of the latent variables of the VRFCAT, among problem solving and processing speed [45]. A decline in working memory has been reported in both AD and schizophrenia [46,47]. Working memory also declines as part of the normal aging process [48].

The ability of VStore to engage cognitive domains implicated in neuropsychiatric disorders and age-related cognitive decline points to its potential in assessing functional cognition not only in healthy individuals but also in clinical populations. In this study, the total time spent in VStore increases with age; hence, age is a significant predictor of most VStore outcomes. However, this may partly be attributed to decrease in technological familiarity with age [49], which could also play a significant role in the outcome of digital assessments. Indeed, ridge regression revealed that the main VStore outcomes—Recall, Find, Select, Pay, and Coffee—provide a parsimonious model and can predict age accurately. Although we cannot make a direct comparison between VStore and Cogstate models, it is observed that Cogstate has a larger slope deviation from the identity line than VStore. Intriguingly, although the inclusion of technological familiarity made VStore model less precise, it did not alter the overall results. In contrast, the Cogstate model was markedly improved by the addition of technological familiarity. This may be because of the additional technological demands of the VStore setup, despite the intuitive nature of the task. The fact that the addition of technological familiarity did not improve the VStore model could be because the variance associated with technological skills was already captured, whereas for Cogstate, this was not the case. As technological familiarity decreased with age, we cannot rule out that VStore, similar to any other digital assessment, may potentially underestimate the cognitive abilities of older adults. As VR tools become more familiar, this relationship may reduce over time, and thus we recommend the assessment of technological familiarity in studies that include participants where these skills may vary.

Similar to these findings, receiver operating characteristic curve analysis revealed that VStore is highly accurate, sensitive, and specific to the classification of age cohorts, further supporting its potential use in the assessment of age-related cognitive decline. This is in line with previous research showing that age is a relevant factor in performance on VR assessments [49,50], potentially explicable by the decline in exploratory navigational abilities—a domain particularly vulnerable to the effects of aging [51]. Effective exploration and navigation are vital for completing VStore and are likely to engage relevant brain regions. Indeed, a key aim in designing the VStore Find task was to activate the place and grid cells in the hippocampus and entorhinal cortex [52]. Notably, this variable was the most strongly associated with increasing age, suggesting that spatial processing, as assessed by VStore, could be used to inform future normative data to detect below-average performance for specified age brackets with high sensitivity.

### Limitations

There are several limitations to this study. First, the study sample had a high IQ on average, as expected from our highly educated cohort; hence, the sample may not be fully representative of the general population. This may be due to an oversampling from college students and a better-educated general population, and the use of the abbreviated IQ measure that relies on only 2 domains, verbal ability and matrix reasoning, and may generate inflated scores [53]. Nonetheless, we were able to include a range of IQ scores. Furthermore, relying on the AIC stepwise algorithm for model selection is not ideal, as it may be affected by several factors, such as the degree of correlation between predictors or the size of the sample, and thus may not be fully replicable [54]. Although theory-based model selection is preferable, given the novelty of the VR task, this was not possible on this occasion. In addition, although ridge regression models were cross-validated by optimizing *λ*, these models should be validated in an independent sample. Future research should also include measures of adverse VR effects; however, it is important to note here that no participant stopped the VR assessment because of cybersickness. Similarly, although there has been no functional capacity assessment developed for healthy adults, the inclusion of a proxy measure, such as the Cognitive Failures Questionnaire [55], would have been desirable. Finally, further research is required to confirm the construct validity of VStore and, most importantly, establish its test-retest reliability.

### Conclusions

In conclusion, our findings suggest that VStore is a promising assessment that engages various cognitive functions, including those that tend to decline with age and during the development of neuropsychiatric disorders such as AD. Given that VStore simulates the complexity of everyday life in an ecologically valid environment, it may be suitable for evaluating functional cognition; however, further research is required to confirm this. VStore has theoretical advantages over other tests in being more engaging than traditional pen-and-paper and computerized batteries; it is fully immersive unlike other similar assessments, such as the VRFCAT, potentially increasing a psychological sensation of *being there* in a specific (virtual) surrounding [56], and thus enabling the assessment of real time cognitive and behavioral responses to that environment [57]. Furthermore, VStore provides complete experimental control, unlike the MET. Further research is urgently required to confirm age-related findings (ie, predictive validity in early cognitive decline) and establish its reliability and sensitivity to changes in cognition and functional capacity in both healthy and clinical samples.

## Appendix

Multimedia Appendix 1 VStore courtyard.

Multimedia Appendix 2 VStore minimarket.

Multimedia Appendix 3 VStore shopping list.

Multimedia Appendix 4 VStore checkout.

Multimedia Appendix 5 Equipment specification.

Multimedia Appendix 6 VStore software.

Multimedia Appendix 7 VStore movement parameters.

Multimedia Appendix 8 Cogstate tasks.

Multimedia Appendix 9 Technological Familiarity Questionnaire.

Multimedia Appendix 10 Mean and SD for VStore outcomes.

Multimedia Appendix 11 Mean and SD for Cogstate outcomes.

Multimedia Appendix 12 Bivariate correlations between Cogstate and VStore outcomes.

Multimedia Appendix 13 Linear regression model assumptions for VStore outcomes.

Multimedia Appendix 14 Bivariate correlations between the Technological Familiarity Questionnaire and Cogstate.

Multimedia Appendix 15 VStore ridge regression cross-validation fit without the Technological Familiarity Questionnaire.

Multimedia Appendix 16 Cogstate ridge regression cross-validation fit without the Technological Familiarity Questionnaire.

Multimedia Appendix 17 VStore ridge regression cross-validation fit with the Technological Familiarity Questionnaire.

Multimedia Appendix 18 Cogstate ridge regression cross-validation fit with the Technological Familiarity Questionnaire.

## Abbreviations

AD: Alzheimer disease
AIC: Akaike Information Criterion
CPAL: Continuous Paired Associate Learning
DET: Detection
DV: dependent variable
GMLT: Groton Maze Learning Task
IDN: Identification
ISLT: International Shopping List Task
IV: independent variable
MET: Multiple Errands Test
MSE: mean squared error
OCL: One Card Learning
ONB: One-Back
TFQ: Technological Familiarity Questionnaire
TWO: Two-Back
VR: virtual reality
VRFCAT: Virtual Reality Functional Capacity Assessment Tool
