# Supplementary figures and images for: A Novel Virtual Reality Assessment of Functional Cognition: Validation Study
Source: J Med Internet Res. 2022 Jan 26;24(1):e27641. doi: 10.2196/27641 (PMC8829700; doi:10.2196/27641)

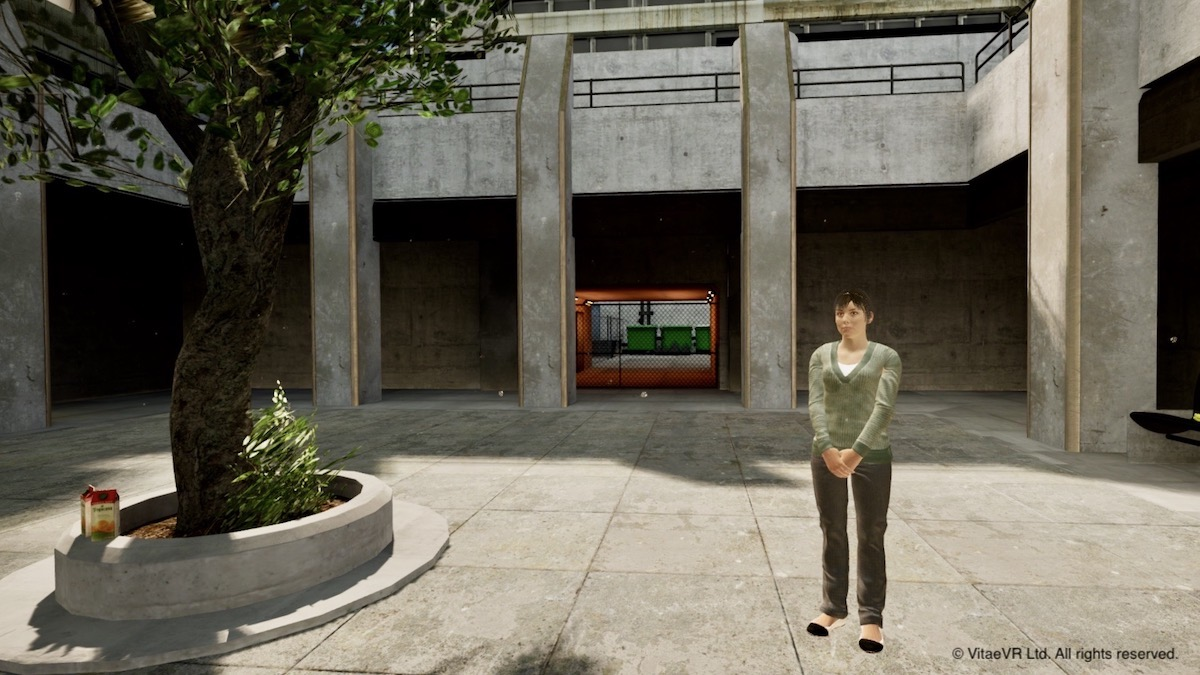

Supplement: Multimedia Appendix 1 [file jmir_v24i1e27641_app1.png]

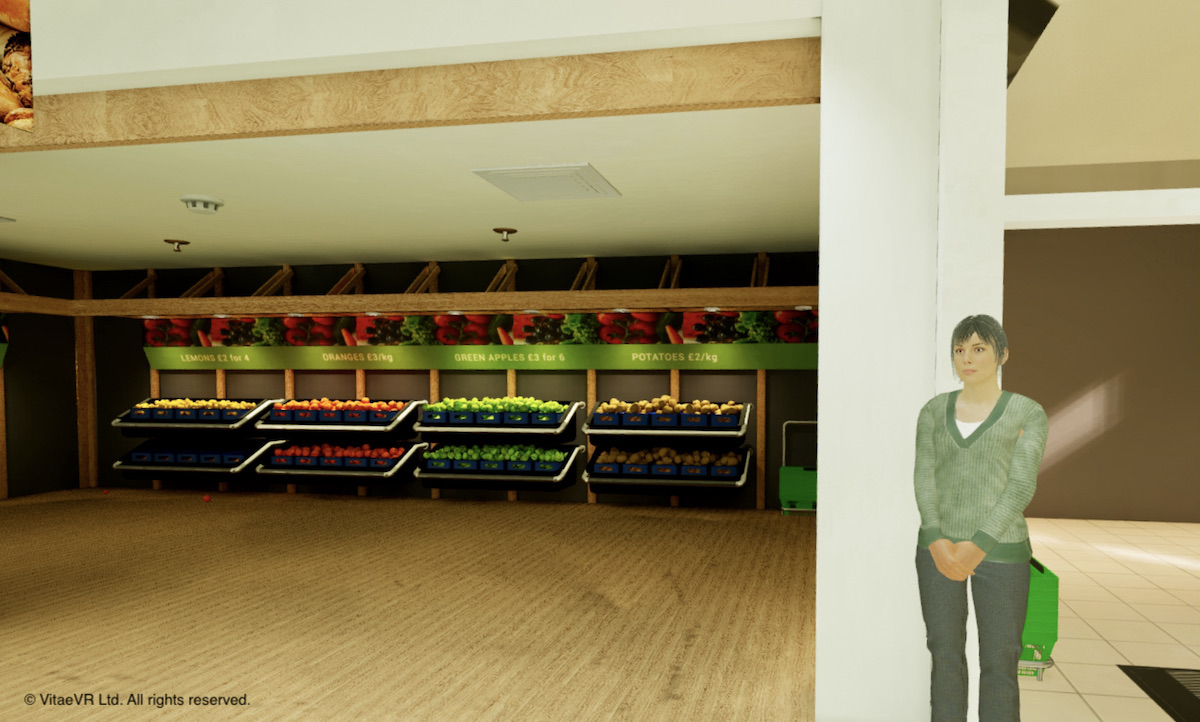

Supplement: Multimedia Appendix 2 [file jmir_v24i1e27641_app2.png]

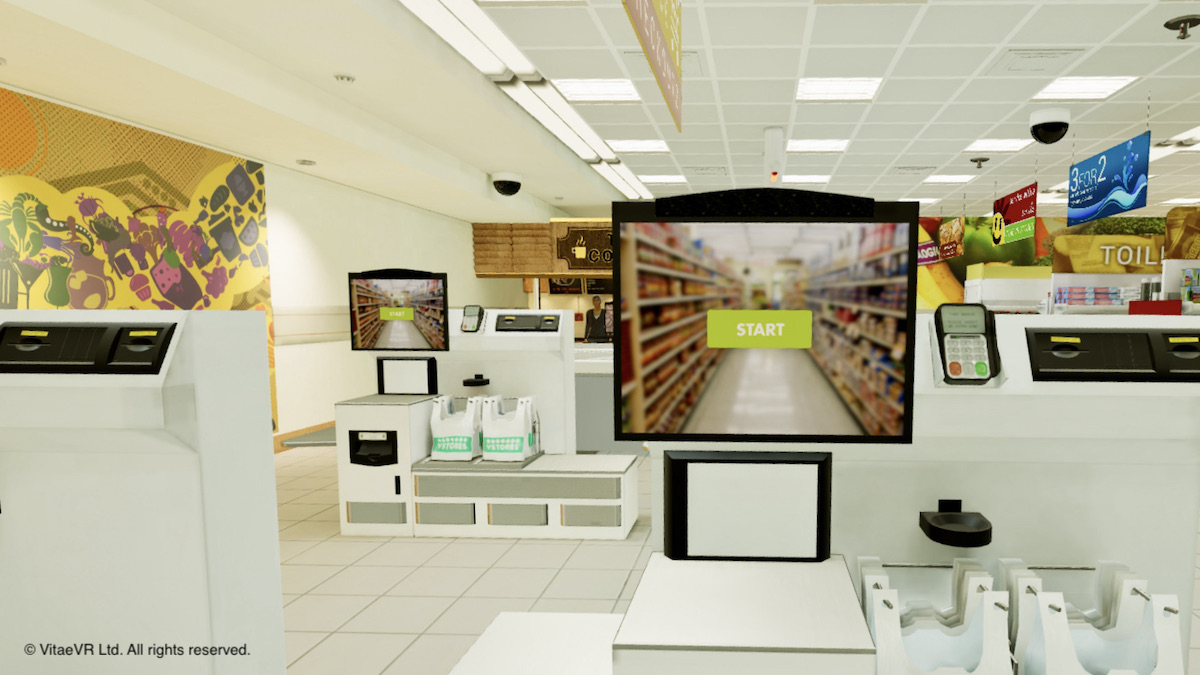

Supplement: Multimedia Appendix 4 [file jmir_v24i1e27641_app4.png]

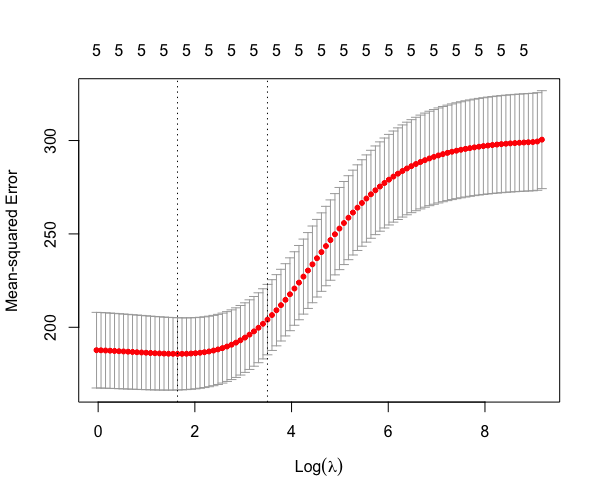

Supplement: Multimedia Appendix 15 [file jmir_v24i1e27641_app15.png]

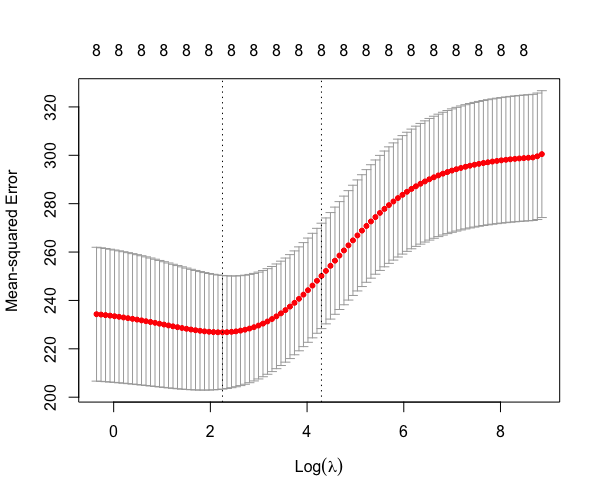

Supplement: Multimedia Appendix 16 [file jmir_v24i1e27641_app16.png]

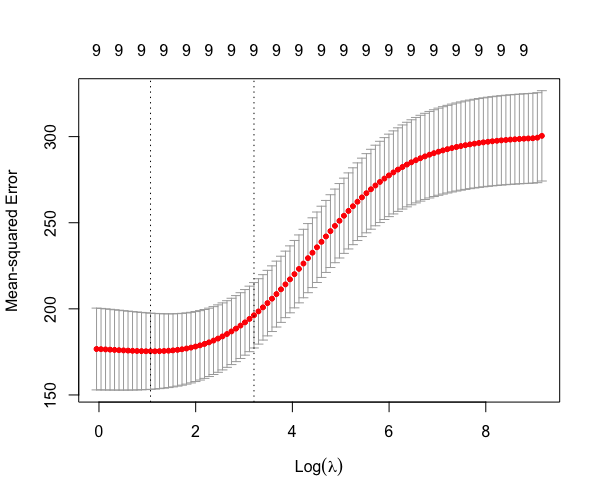

Supplement: Multimedia Appendix 17 [file jmir_v24i1e27641_app17.png]

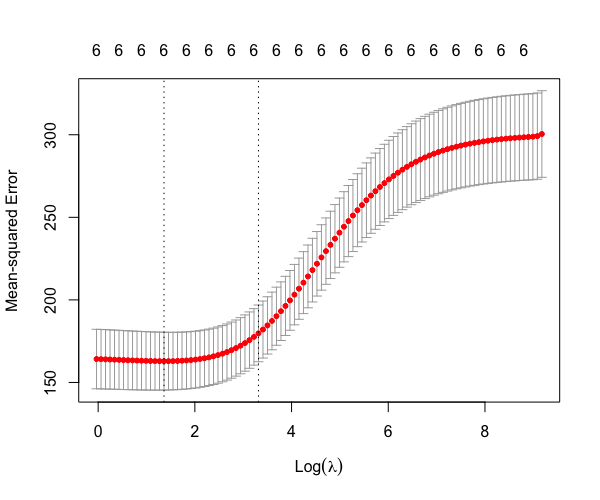

Supplement: Multimedia Appendix 18 [file jmir_v24i1e27641_app18.png]
