# Supplementary material for: A Novel Virtual Reality Assessment of Functional Cognition: Validation Study
Source: J Med Internet Res. 2022 Jan 26;24(1):e27641. doi: 10.2196/27641 (PMC8829700; doi:10.2196/27641)
Supplement: Multimedia Appendix 3 [file jmir_v24i1e27641_app3.docx]

**Multimedia Appendix** **4.** VStore shopping list.

| 1. | Cornflakes | 7. | Colgate Toothpaste |
| --- | --- | --- | --- |
| 2. | Tropicana Orange Juice | 8. | Red Apple |
| 3. | Coca Cola | 9. | Lemon Curd |
| 4. | Full Fat Milk | 10. | Potato |
| 5. | Tuna Sandwich | 11. | Orange |
| 6. | Head and Shoulders | 12. | Brown Bread |
