# Supplementary material for: A Novel Virtual Reality Assessment of Functional Cognition: Validation Study
Source: J Med Internet Res. 2022 Jan 26;24(1):e27641. doi: 10.2196/27641 (PMC8829700; doi:10.2196/27641)
Supplement: Multimedia Appendix 5 [file jmir_v24i1e27641_app5.docx]

**Multimedia Appendix** **5.** Equipment specification.

In this study, the software was run in SteamVR on an Alienware Area-51 R2 PC (Processor: Intel® Core™ i7-6800K CPU 3.40 GHz, RAM: 32GB, Video Card: Nvidia TITAN X Pascal, OS: Microsoft Windows 10 Pro). The VR environment was displayed using HTC VIVE™ ([www.vive.com](http://www.vive.com)), including a head-mounted display (HDM), two controllers, two tracking sensors, and a link box to connect the headset to the computer via HDMI and USB 2.0 cables. The HMD has a Dual AMOLED 3.6″ diagonal screen with resolution of 1080 x 1200 pixels per eye (2160 x 1200 pixels combined), refresh rate of 90 Hz, and field of view of 110˚. This set-up allows for complete immersion, providing a 360° first-person view of the virtual environment.
