# Supplementary material for: A Novel Virtual Reality Assessment of Functional Cognition: Validation Study
Source: J Med Internet Res. 2022 Jan 26;24(1):e27641. doi: 10.2196/27641 (PMC8829700; doi:10.2196/27641)
Supplement: Multimedia Appendix 6 [file jmir_v24i1e27641_app6.docx]

**Multimedia Appendix 6.** VStore software.

VStore was developed using Unreal Engine 4 (UE4) written in C++ programming language. It was originally developed for Microsoft Windows operating system; but since has been adapted to PlayStation 4, and could potentiality be adapted to other platforms. VStore is a propriety software developed in collaboration by Vitae VR Ltd and King’s College London.
