# Supplementary material for: A Novel Virtual Reality Assessment of Functional Cognition: Validation Study
Source: J Med Internet Res. 2022 Jan 26;24(1):e27641. doi: 10.2196/27641 (PMC8829700; doi:10.2196/27641)
Supplement: Multimedia Appendix 7 [file jmir_v24i1e27641_app7.docx]

**Multimedia Appendix 7.** VStore movement parameters.

VStore, using HTC Vive, allows for six degrees of freedom motion tracking including rotational movement around the x, y, and z axes; and translational movement along those axes moving forward or backward, left or right, and up or down. Participants are free to walk around within the environment scaled to the testing room at 3.6 x 3.1 meters. However, the testing space is smaller than the virtual minimarket environment; therefore, participants are also required to use instant teleportation. This involves pressing the trackpad on the controller and aiming at the desired destination, and upon release, the player teleports to that location. This method is instantaneous and does not include animation that explains the movement. While instant teleportation may reduce ecological validity, it also reduces the likelihood of inducing cybersickness.
