# Supplementary material for: A Novel Virtual Reality Assessment of Functional Cognition: Validation Study
Source: J Med Internet Res. 2022 Jan 26;24(1):e27641. doi: 10.2196/27641 (PMC8829700; doi:10.2196/27641)
Supplement: Multimedia Appendix 8 [file jmir_v24i1e27641_app8.pdf]

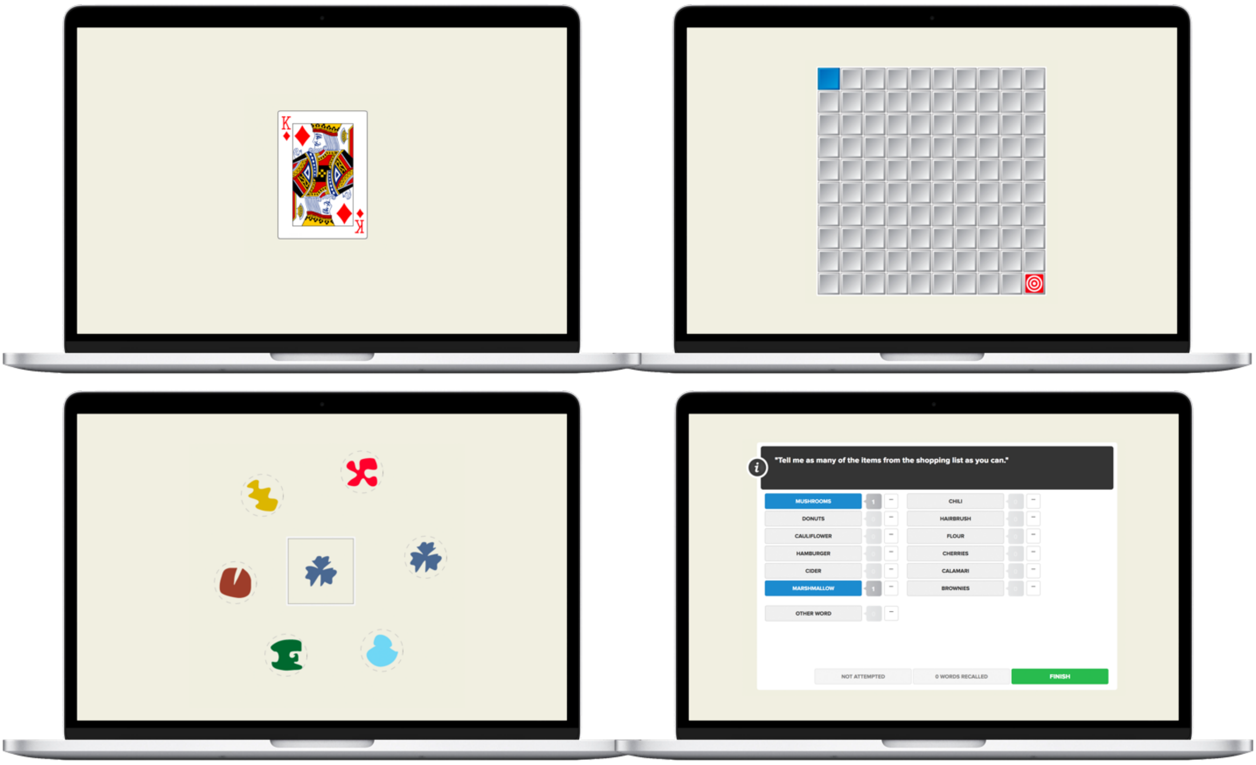
"Tell me as many of the items from the shopping list as you can."
MUSHROOMS
1
CHILI
DONUTS
HAIRBRUSH
CAULIFLOWER
FLOUR
HAMBURGER
CHERRIES
CIDER
CALAMARI
MARSHMALLOW
1
BROWNIES
OTHER WORD
NOT ATTEMPTED
0 WORDS RECALLED
FINISH
