# Supplementary material for: A Novel Virtual Reality Assessment of Functional Cognition: Validation Study
Source: J Med Internet Res. 2022 Jan 26;24(1):e27641. doi: 10.2196/27641 (PMC8829700; doi:10.2196/27641)
Supplement: Multimedia Appendix 9 [file jmir_v24i1e27641_app9.docx]

**Multimedia Appendix 9.** Technological Familiarity Questionnaire.

The first question consists of six sub-items ascertaining how frequently participants use 1) a computer and 2) a smartphone; play video games on 3) a computer, 4) smartphone, and 5) console (e.g. PS4); and 6) use VR. The second question also consists of six sub-items measuring how comfortable participants are with using 1) a computer and 2) a smartphone; playing video games on 3) a computer, 4) smartphone, and 5) console; and 6) using VR. The third question consists of a single item measuring overall ability to use technology.
