# Supplementary material for: A Novel Virtual Reality Assessment of Functional Cognition: Validation Study
Source: J Med Internet Res. 2022 Jan 26;24(1):e27641. doi: 10.2196/27641 (PMC8829700; doi:10.2196/27641)
Supplement: Multimedia Appendix 10 [file jmir_v24i1e27641_app10.docx]

**Multimedia Appendix** **10.** Means and standard deviations (SD) for VStore outcomes.

| Group | Recall N  (SD) | Find Sec  (SD) | Select Sec (SD) | Pay Sec  (SD) | Coffee Sec (SD) | Total Sec (SD) |
| --- | --- | --- | --- | --- | --- | --- |
| 20-29 | 7.1 (1.8) | 5.90 (0.26) | 4.72 (0.33) | 3.02 (0.39) | 3.22 (0.32) | 6.27 (0.24) |
| 30-39 | 6.9 (1.9) | 6.05 (0.24) | 4.68 (0.38) | 2.97 (0.35) | 3.35 (0.52) | 6.38 (0.20) |
| 40-49 | 6.9 (1.7) | 6.10 (0.24) | 4.77 (0.32) | 3.10 (0.41) | 3.35 (0.50) | 6.45 (0.23) |
| 50-59 | 6.0 (1.7) | 6.23 (0.30) | 4.96 (0.21) | 3.13 (0.42) | 3.76 (0.32) | 6.58 (0.25) |
| 60-69 | 6.5 (1.7) | 6.27 (0.21) | 4.97 (0.22) | 3.35 (0.35) | 3.86 (0.39) | 6.63 (0.18) |
| 70-79 | 5.4 (1.7) | 6.45 (0.26) | 5.09 (0.22) | 3.45 (0.38) | 3.85 (0.39) | 6.78 (0.22) |
