# Supplementary material for: A Novel Virtual Reality Assessment of Functional Cognition: Validation Study
Source: J Med Internet Res. 2022 Jan 26;24(1):e27641. doi: 10.2196/27641 (PMC8829700; doi:10.2196/27641)
Supplement: Multimedia Appendix 11 [file jmir_v24i1e27641_app11.docx]

**Multimedia Appendix** **11.** Means and standard deviations (SD) for Cogstate outcomes.

| Group | DET  (SD) | IDN  (SD) | OCL  (SD) | ONB  (SD) | TWO (SD) | GMLT (SD) | CPAL (SD) | ISLT  (SD) |
| --- | --- | --- | --- | --- | --- | --- | --- | --- |
| 20-29 | 2.44 (0.04) | 2.65 (0.05) | 1.06 (0.08) | 2.80 (0.09) | 1.30 (0.13) | 43.42 (19.51) | 72.89 (60.91) | 28.63 (4.26) |
| 30-39 | 2.50 (0.08) | 2.65 (0.06) | 1.05 (0.08) | 2.83 (0.08) | 1.30 (0.19) | 42.61 (16.22) | 51.22 (52.85) | 26.72 (4.31) |
| 40-49 | 2.49 (0.06) | 2.70 (0.05) | 1.00 (0.09) | 2.86 (0.07) | 1.29 (0.16) | 53.61 (19.55) | 102.89 (44.56) | 26.67 (3.82) |
| 50-59 | 2.49 (0.09) | 2.67 (0.07) | 1.05 (0.08) | 2.85 (0.08) | 1.28 (0.16) | 53.53 (20.57) | 113.94 (71.34) | 26.35 (3.50) |
| 60-69 | 2.54 (0.08) | 2.70 (0.04) | 1.03 (0.07) | 2.87 (0.06) | 1.24 (0.12) | 52.56 (19.25) | 98.78 (70.33) | 26.28 (2.74) |
| 70-79 | 2.53 (0.09) | 2.71 (0.04) | 1.02 (0.07) | 2.92 (0.06) | 1.26 (0.12) | 61.57 (24.31) | 133.86 (42.59) | 21.93 (3.65) |
