# Supplementary material for: A Novel Virtual Reality Assessment of Functional Cognition: Validation Study
Source: J Med Internet Res. 2022 Jan 26;24(1):e27641. doi: 10.2196/27641 (PMC8829700; doi:10.2196/27641)
Supplement: Multimedia Appendix 12 [file jmir_v24i1e27641_app12.docx]

**Multimedia Appendix** **12.** Bivariate correlations between Cogstate and VStore outcomes.

|  |  | VStore Recall | VStore  Find | VStore Select | VStore  Pay | VStore Coffee | VStore Total |
| --- | --- | --- | --- | --- | --- | --- | --- |
| Processing Speed  (DET) | Corr.  Coef. | 0 | 0.3 | 0.2 | 0.4 | 0.3 | 0.3 |
|  | Sig (2-tailed) | 1.00 | .02 | .25 | <.001 | .07 | .004 |
| Attention  (IDN) | Corr.  Coef. | –0.1 | 0.2 | 0.1 | 0.4 | 0.2 | 0.2 |
|  | Sig (2-tailed) | 1.00 | .30 | 1.00 | .001 | .21 | .09 |
| Visual Learning  (OCL) | Corr.  Coef. | 0.2 | –0.2 | –0.2 | –0.1 | 0 | –0.2 |
|  | Sig (2-tailed) | 1.00 | .77 | .42 | 1.00 | .01 | .50 |
| Working Memory  (ONB) | Corr.  Coef. | –0.2 | 0.4 | 0.3 | 0.5 | 1.00 | 0.4 |
|  | Sig (2-tailed) | .59 | <.001 | 0.2 | <.001 | .01 | <.001 |
| Working Memory  (TWO) | Corr.  Coef. | 0.2 | –0.3 | –0.3 | –0.4 | –0.3 | –0.3 |
|  | Sig (2-tailed) | .19 | .02 | .01 | <.001 | .03 | .003 |
| Executive Functions  (GMLT) | Corr.  Coef. | –0.1 | 0.4 | 0.4 | 0.4 | 0.2 | 0.4 |
|  | Sig (2-tailed) | 1.00 | .001 | .001 | <.001 | .15 | <.001 |
| Paired Associate Learning  (CPAL) | Corr.  Coef. | –0.2 | 0.4 | 0.4 | 0.3 | 0.3 | 0.5 |
|  | Sig (2-tailed) | .64 | <.001 | .001 | .01 | .01 | <.001 |
| Verbal Learning  (ISLT) | Corr.  Coef. | 0.4 | –0.2 | –0.4 | –0.3 | –0.3 | –0.3 |
|  | Sig (2-tailed) | <.001 | .10 | .001 | .01 | .01 | .002 |
