# Supplementary material for: A Novel Virtual Reality Assessment of Functional Cognition: Validation Study
Source: J Med Internet Res. 2022 Jan 26;24(1):e27641. doi: 10.2196/27641 (PMC8829700; doi:10.2196/27641)

Multimedia Appendix 13. Linear regression model assumptions for VStore outcomes.

A. VStore Recall


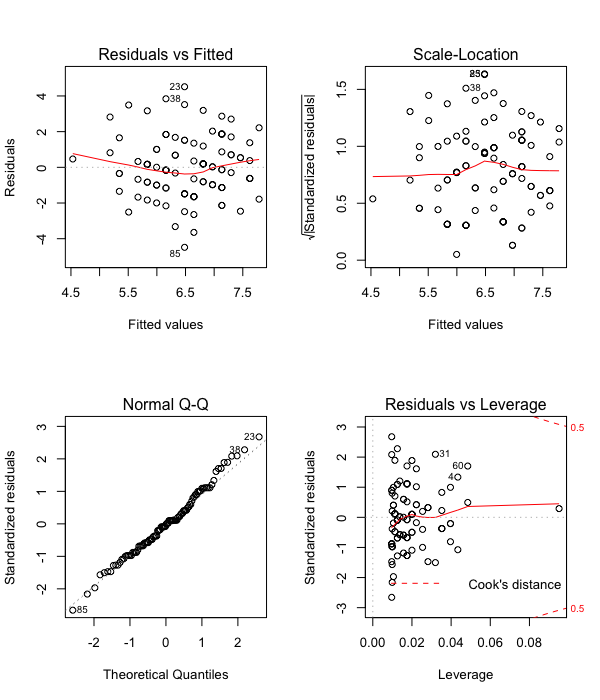


B. VStore Find


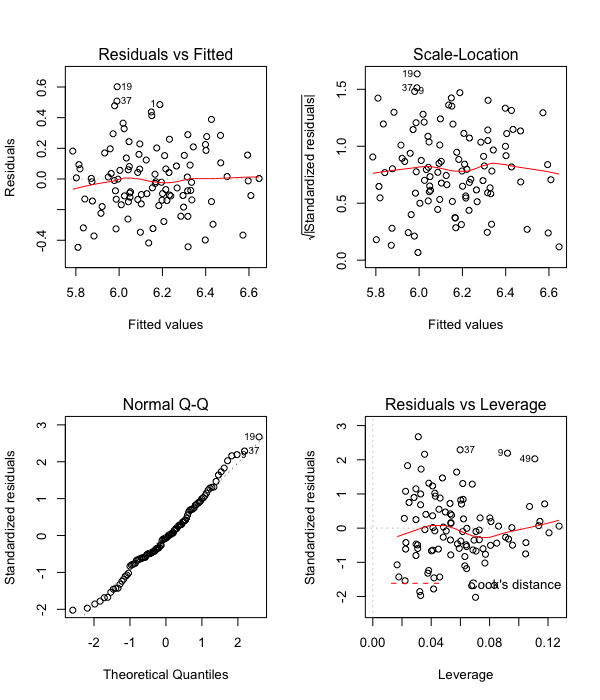


C. VStore Select
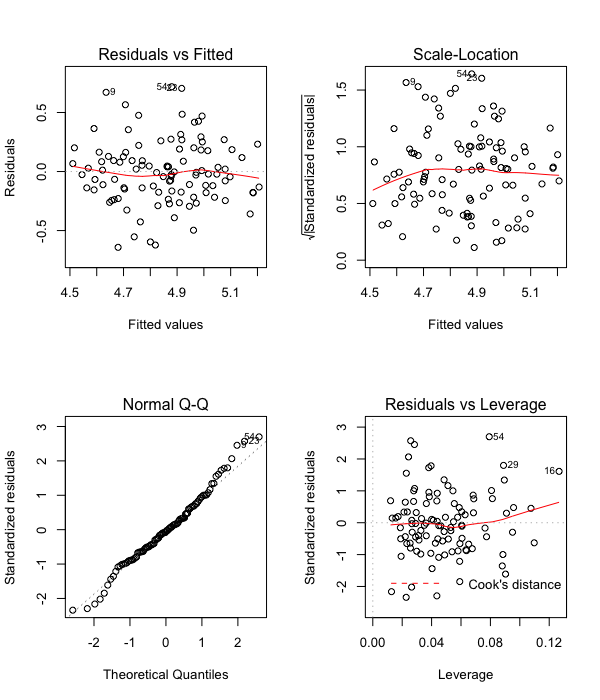


D. VStore Pay
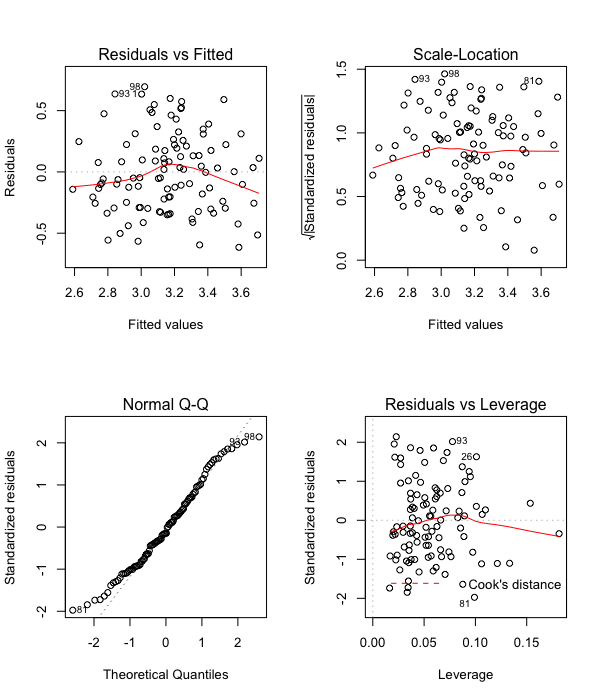


E. VStore Coffee
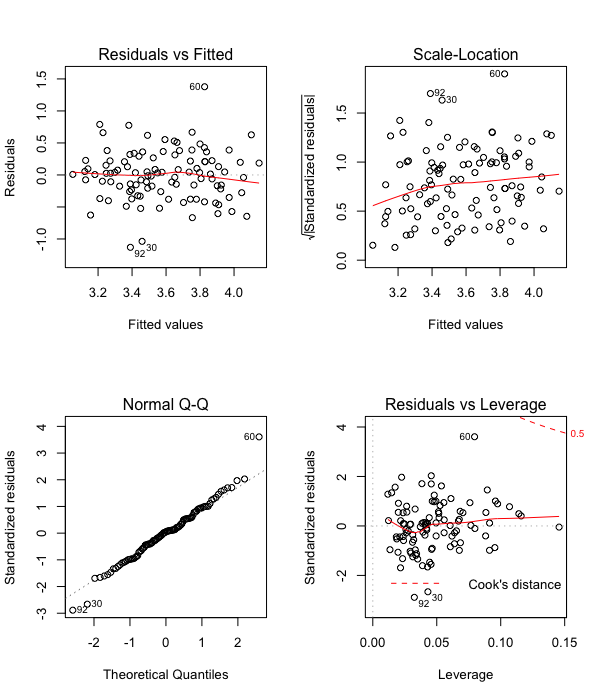


F. VStore Total


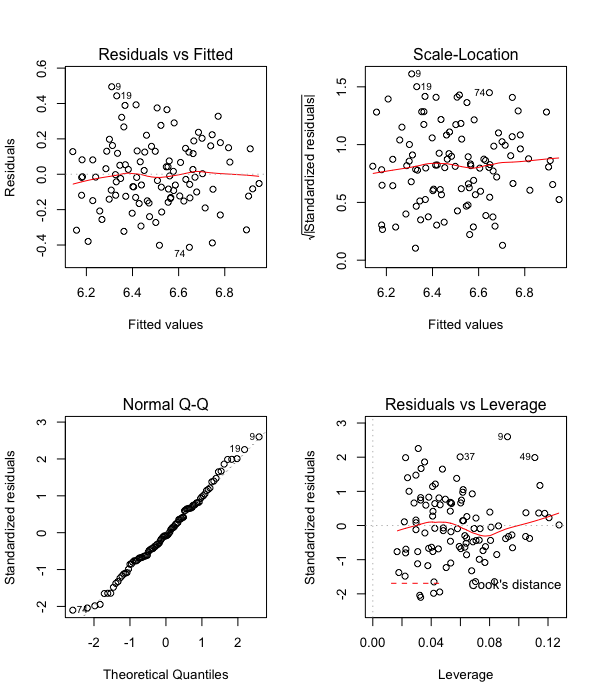

Supplement: Multimedia Appendix 13 [file jmir_v24i1e27641_app13.docx]
