# Supplementary material for: A Novel Virtual Reality Assessment of Functional Cognition: Validation Study
Source: J Med Internet Res. 2022 Jan 26;24(1):e27641. doi: 10.2196/27641 (PMC8829700; doi:10.2196/27641)
Supplement: Multimedia Appendix 14 [file jmir_v24i1e27641_app14.docx]

**Multimedia Appendix** **19.** Bivariate correlations between the TFQ and Cogstate.

|  | DET | IDN | OCL | ONB | TWO | GMLT | CPAL | ISLT |
| --- | --- | --- | --- | --- | --- | --- | --- | --- |
| TFQ | –0.3^***^ | –0.3^***^ | 0.0 | –0.4^***^ | 0.3^***^ | –0.2^*^ | –0.3^***^ | 0.0 |
| Spearman’s rho, Significance level. ^*^p ≤ 905; ^**^p ≤ 901; ^***^p ≤ 9001 | | | | | | | | |
